# A randomized placebo-controlled trial of the efficacy and safety of a terbinafine, florfenicol and betamethasone topical ear formulation in dogs for the treatment of bacterial and/or fungal otitis externa

**DOI:** 10.1186/s12917-018-1589-7

**Published:** 2018-08-31

**Authors:** S. L. Forster, T. Real, K. P. Doucette, S. B. King

**Affiliations:** 1 Elanco Animal Health, 2500 Innovation Way, Greenfield, IN 46140 USA; 2 Elanco Animal Health Ltd., Lilly House, Priestley Road, Basingstoke, Hampshire, RG24 9NL UK

**Keywords:** Otitis externa, Otic gel, Clinical efficacy, Clinical safety, Florfenicol, Bethamethasone, Terbinafine

## Abstract

**Background:**

Treatment of infected otitis externa (OE) relies on the topical application of specific formulations that most often contain an antibiotic, an antifungal and a glucocorticoid. This study is to report the results of a randomized, placebo-controlled field trial evaluating the efficacy and safety of OSURNIA™ (Elanco Animal Health, a division of Eli Lilly and Company, Greenfield, IN), a novel topical ear medication containing florfenicol, terbinafine and betamethasone acetate in an adaptable gel. The study includes 284 dogs with bacterial and/or fungal OE who were randomly assigned to receive two doses of Osurnia or its vehicle, one week apart. Dogs were evaluated at various time points through Day 45, and a total clinical score (TCS) was calculated based on pain, erythema, exudate, swelling, odor and ulceration. The primary outcome measure was the rate of treatment success (RTS), defined as a TCS of 0, 1 or 2 on Day 45. Before and after treatment, a “clap test” was performed to subjectively assess hearing, and blood and urine were collected for routine clinical pathology.

**Results:**

The RTS was significantly higher in ears treated with Osurnia (64.78%) than with placebo (43.42%). There was no significant interaction between efficacy and duration of history, recurrence of otitis or body weight. Adverse events were similar between groups. All dogs treated with Osurnia maintained their hearing, and there were no relevant clinical pathology changes.

**Conclusions:**

The application of two doses of Osurnia, one week apart, is effective and safe to treat microbial otitis externa in dogs.

## Background

Otitis externa is one of the most common medical conditions affecting dogs. In one epidemiological study of more than 2300 canine general practice consultations, a dermatological problem (21%) was the second most frequent reason motivating owners to consult their veterinarians after preventive veterinary care [1]. Among dogs with skin ailments, OE was the third most common diagnosis given (22% of patients) [1]. Similarly, in a more recent analysis of a random sample of more than 3500 primary care electronic medical records, OE was confirmed to be the most frequently made diagnosis in dogs (10%) [2].

Otitis externa is a syndrome characterized by inflammation of the external ear canal; it is traditionally attributed to a combination of predisposing (e.g. ear canal conformation, moisture, etc.), primary (e.g. allergic, parasitic, foreign bodies, etc.), perpetuating (e.g. hyperplasia, stenosis, etc.) and secondary (e.g. bacteria, yeast infections, etc.) factors [3]. Indeed, commensal microbes from the external ear canal of normal dogs can proliferate—often because of the underlying factors listed above—and infections with pathogens such as *Staphylococcus pseudintermedius, Pseudomonas aeruginosa, Proteus mirabilis* and *Malassezia pachydermatis* appear to be the dominant problem [4, 5]. The impact of OE is notable, as otitis diminishes the quality of life of dogs and their owners [6].

Although there are no standard guidelines for treatment of canine OE, veterinarians have long used topical formulations developed for this indication; these medications usually include a broad-spectrum antibiotic, an antifungal and a glucocorticoid to reduce inflammation in an attempt to rapidly decrease pain and discomfort [7, 8]. Although it is considered good practice to evaluate results of ear cytology and microbial cultures, which, unfortunately, can yield discordant results [9], the selection of a topical ear formulation in general practice appears more often influenced by subjectivity, prescription habits, and commercial aspects of product supply [7].

The application of topical ear formulations to dogs with OE is fraught with difficulties that can decrease owner adherence to a treatment regimen. The need for once or twice daily drug application into painful and/or stenotic ear canals can be stressful for owners and lead to aggressive behaviours from dogs [6]. Furthermore, in multidose presentations the repeated placement of the tip of the product into infected ear canals could potentially lead to their mutual microbial cross-contamination similar to may be seen with otoscope tips [4]. Failure of compliance can reduce treatment efficacy, but it might also promote the development of bacterial resistance to antibiotics [5]. As a result of these limitations, a one-time use flexible applicator applied less frequently would more than likely improve treatment compliance.

Osurnia (Elanco Animal Health, Greenfield, IN, USA) is a novel topical ear therapy formulated as a pre-measured (1 mL), easy-to-use, single-dose tube with a flexible soft tip; it contains the antibiotic florfenicol (10 mg/mL), the antifungal terbinafine (10 mg/mL) and the glucorticoid betamethasone acetate (1.0 mg mL) in an adaptable gel vehicle designed to coat the ear canal and assist with product remaining where applied.

The objective of this article is to report the results of a randomized controlled trial (RCT) evaluating the efficacy and safety of the novel topical ear product Osurnia, administered twice per affected ear, one week apart, to dogs with bacterial and/or fungal OE. This report aimed at strictly following the 2010 Consolidated Standards of Reporting Trials (CONSORT) guidelines, an evidence-based, minimum set of recommendations for reporting randomized trials (http://www.consort-statement.org; page last accessed January 17, 2018).

## Methods

### Trial design

This trial was a 6-week randomized double-blinded placebo (vehicle)-controlled multicenter parallel trial; the study followed the US FDA’s Good Clinical Practices guideline VICH GL9.

### Study participants

Dogs of any gender, weight, breed and of at least 8 weeks of age with OE were considered for enrollment after their owner gave informed consent for their dog to participate in this RCT.

To be enrolled in the study, the presence of an active OE was determined by a TCS of 6 or more based on the clinical assessment (including otoscopy) and scoring of the following: pain, erythema, exudate, swelling, odor and ulceration (Table 1).

**Table 1:** Total clinical score

Clinical sign	Score ‘0’ descriptor	Score ‘1’ descriptor	Score ‘2’ descriptor
Pain	None noted	Mild/moderate; painful on palpation	Severe: painful when raising pinna
Erythema	None noted	Mild/moderate: barely perceptible to obvious redness visible with an otoscope	Severe: beet or cherry red or erythema extends into pinna
Exudate	None noted	Mild/moderate: small amount to moderate amounts visible in ear canal	Severe: extending out of ear canal and may be crusted
Swelling	None noted	Mild/moderate: minimal occlusion to moderate occlusion of ear canal	Severe: canal completely occluded
Odor	None noted	Mild/moderate: malodor evident when pinna is raised	Severe: malodor evident without raising pinna to expose ear canal
Ulceration	None noted	Mild/moderate: mild abrasions	Severe: abrasions that may be bleeding

The OE was characterized as “acute” if the history suggested that the signs of otitis had developed within 6 days of the initial visit, “subchronic” if they had occurred within 7 to 30 days prior to the visit or “chronic” if they had been present for more than 30 days beforehand. The OE was deemed to be recurrent if the dog had been successfully treated for a similar problem (i.e. with no remaining signs of OE post-treatment) in the preceding 3 months. Finally, the otitis exudate was described as erythematous/ceruminous or suppurative.

Importantly, before an ear was considered for treatment, its tympanic membrane had to be visible and assessed to be intact. After scoring on Day 0, exudate was removed using saline in order to allow visualization of the tympanic membrane. Cleaning was not repeated at Day 7.

Exclusion criteria for this RCT included dogs that had been treated beforehand with systemic, topical or otic antibiotics or antifungals within 14 days, systemic or topical anti-inflammatory drugs—including short-acting glucocorticoids—for 14 days, long-acting glucocorticoids for 28 days, cyclosporine for 14 days, otic ear cleaners for 7 days, antihistamines for 14 days and analgesics for 7 days. An *Otodectes cynotis* infestation or an otic foreign body were both considered exclusion criteria. Finally, dogs with known or suspected hypersensitivity to Osurnia’s active ingredients (florfenicol, terbinafine or betamethasone acetate), dogs intended for breeding, pregnant or lactating bitches, staff-owned pets and those enrolled in other clinical trials were not considered further.

Dogs were selected from 15 companion animal general practices located in various geographic locations in the USA; this wide selection being done to adequately represent the targeted population for the product being developed. Each site was required to enroll a minimum of 4 dogs (2 evaluable dogs per treatment group) and was not allowed to exceed 30 dogs.

After having been treated with one of the test products, dogs could be removed from the trial for any of the following reasons:

- the presence of a serious adverse event (AE);
- a lack of improvement in TCS on Days 7, 14 or 30, which would justify the need for conventional therapy;
- the perception of lack of test product efficacy at any study day; or
- the administration of, or perceived need for, forbidden concomitant therapy (see below).

In any of these situations, the reasons for withdrawal were documented.

### Interventions

Before the first administration of the test products, the ear canals to be treated were cleaned with saline to remove exudate and enable the verification of an intact tympanum. Ears were not flushed prior to the second administration to maintain treatment in the affected ear.

The intervention consisted of the topical application of 1 mL of the active product (Osurnia) or its vehicle (placebo control), once on Day 0 and a second time on Day 7 (±2 days). Either one of the two test products was applied into the ear canal followed by a massage at the base of the ear to allow for its homogeneous distribution. If both ears were affected, treatment was permitted for both, but only the data from the right ear were collected and evaluated for this study, provided that its initial TCS was 6 or greater. The same test product was used to treat both ears.

Concomitant otic medications were not allowed until the exit of the patient from this trial. Additionally, the use of systemic or topical antibiotics, antifungals, anti-inflammatory drugs, glucocorticoids, cyclosporine, analgesics and antihistamines were not permitted until study end. Dogs removed early during the study due to perceived lack of efficacy would be considered as treatment failures, and the investigator would clean the ear(s) with saline and use conventional otitis treatment.

### Sample size

Anticipated recruitment was 225 dogs with OE for inclusion to reach a minimum of 150 evaluable cases (i.e. animals which fulfilled the requirements for per-protocol evaluation; assessment of eligibility for inclusion into the efficacy evaluation was determined without knowledge of treatment administered) in a 2:1 ratio of active-versus-vehicle (i.e. 100 dogs treated with Osurnia and 50 administered placebo). Based on prior pilot study results, this number of dogs was calculated to give this study an approximate 85% power to detect a treatment success of 70% with Osurnia and 45% with the placebo.

### Randomization

Dogs included in this study were assigned to one of the two treatment groups in a 2:1 ratio according to a randomization sequence generated by computer by the Sponsor’s statistician using the STAT version 9 software (SAS, Cary, NC, USA). Each study site had a separate randomization schedule in which dogs were blocked in groups of six.

The original study randomization code was sealed in an envelope and kept by the study sponsor until the end of the study. At each veterinary clinic site, one individual was given the role of “*dispenser*”: this person assigned each successively enrolled dog to a letter (A, B, C, D, E or F) corresponding to one of the two treatment groups according to the randomization schedule, and he/she administered the active or placebo ear medications to the patients.

### Blinding

At each veterinary clinic, all personnel (including the dispenser) were masked to treatment; the pet owner was also blinded to the intervention. To further preserve this blinding, the dispenser was asked not to disclose any treatment related information to the investigator responsible for grading the effect of treatment. Additionally, the dispenser was not allowed to gather or interpret any data.

Masking was done by placing the active medication (Osurnia) and its vehicle control into identical tubes and packages labeled with one of six code letters (A to F) corresponding to a block of six dogs. Only select study Sponsor personnel knew the treatment associated with each letter code, and it was not made available to study monitors, the statistician and clinical safety representative until the end of the trial prior to final report compilation. There were no reports of unblinding at any participating site.

### Participant evaluation

Dogs were examined on Days 0, 7, 14, 30 and 45 or during an exit visit in case of early withdrawal. Clinical examinations included a routine assessment of general appearance and major systems (cardiological, ocular, auricular, gastrointestinal, neurological, respiratory, urogenital, etc.). Pre-existing medical conditions were also recorded. Additional clinical examinations were to be performed in case of a serious AE. Body weights were collected and recorded in pounds rounded to the nearest 0.1 lb.

At each visit, the investigator performed an otoscopic examination and used a standard score to grade the presence or absence of pain, erythema, exudate, swelling, odor and ulceration in the ear canal of the evaluable ear (predetermined as the right ear if both ears were affected and qualified). These observations were then compared to those before treatment, and they were scored as “*no increase in irritation*” or “*increase in irritation*”.

Finally, a subjective evaluation of the dog’s ability to hear was measured by a “*clap test*” before treatment (Day 0) and on Day 45 or earlier in case of premature study withdrawal. Personnel at the study site clapped their hands together in a location outside of the dog’s field of vision and the ability of the dog to turn its head toward the noise was observed. The following recordings were made: “*dog can hear*” or “*dog cannot hear*” and the dog was then assigned to one of the following four categories:

- Dog could hear at Day 0 and at Day 45 (or study exit)
- Dog could hear at Day 0 and could not hear at Day 45 (or study exit)
- Dog could not hear at Day 0 and could hear at Day 45 (or study exit)
- Dog could not hear at Day 0 nor at Day 45 (or study exit)

### Microbial cultures

Before treatment and on Day 45 if a clinical cure (i.e. a TCS of 0, 1 or 2) was not achieved, a swab of the affected ear canal(s) were cultured for bacteria and yeast with testing of their sensitivity to Osurnia’s antimicrobial drugs florfenicol and terbinafine. A similar procedure was also done before Day 45 for any dog removed from the trial due to perceived treatment failure. Evaluated (qualifying) pathogens were *Malassezia pachydermatis*, *Candida albicans*, *Staphylococcus pseudintermedius*, *Pseudomonas aeruginosa*, beta-hemolytic *Streptococcus species*, *Escherichia coli*, and *Proteus mirabilis*. To be considered as an evaluable case for efficacy positive growth of at least one of these qualifying pathogens had to be present in the affected ear. Minimum inhibitory concentrations (MICs)_50/90_ were calculated for florfenicol and terbinafine.

### Outcome measures of efficacy

Veterinarian investigators were responsible for determining the severity of OE in affected ears before and 7, 14, 30 and 45 days after starting topical ear therapy with the tested products using the scoring system and the calculation of a TCS, as shown in Table 1. In case of premature study exit, a TCS was also assessed at the time of withdrawal.

The primary outcome measure for efficacy was the RTS on Day 45, this being the official end of the study. Treatment success was defined as a TCS of 0, 1 or 2 on a scale of 12 at study end. Cases exiting the study early for reasons related to treatment (i.e. the presence of an AE, a lack of perceived efficacy, etc.) were considered treatment failures regardless of their final TCS.

### Safety evaluation

Adverse events were defined as any observation in a study participant that was unfavorable, unintended and that occurred after the use of a test product, whether or not it was considered to be related to the product being administered. A serious AE was defined as one that was considered clinically significant by the investigator and that also required medical intervention. In this RCT, pet owners or study personnel reported all potential AEs as soon as possible to the local investigator; after assessment of the duration, severity, presumed relationship of the event to the administered product and its outcome, AEs were then recorded as “*serious*” or “*not serious*” as defined above. Pre-existing conditions were not considered AEs unless the condition worsened after treatment with the test products.

Before enrollment and on Day 45 or earlier in case of premature study exit, blood and urine were collected for routine hematology, clinical biochemistry and urinalysis (catheterization, free catch or cystocentesis), respectively. Additional samples were collected in case of serious AE occurring during the study.

### Statistical methods

Baseline demographic (age, weight, gender, breeds) and OE data (number of ears treated, type of otitis [acute, subchronic, or chronic], recurrent status, exudate type [suppurative or erythematous ceruminous], and hearing status) were first summarized by treatment groups (Osurnia versus vehicle) using SAS PROC FREQ or SAS PROC MEANS, depending on the type of variable. A T-test was used to compare the mean ages at baseline and Fisher’s Exact tests were used to compare all other variables except for breed.

For efficacy evaluation, the clinical outcome for each case (“*success*” or “*failure*”) was analyzed using SAS PROC GLIMMIX with the main effects of “*treatment*”, “*site*” and the interaction “*treatment by site*”, with “*treatment*” being a fixed effect and the other two being random effects. A logit link function was employed. The Variance Components (VC) covariance structure was utilized and the Containment method was employed to estimate the denominator degrees of freedom. All efficacy variables (erythema, exudate, odor, pain, swelling and ulceration) were summed on Day 45 (or at study exit if an animal exited early) to obtain a TCS. A case was classified as a “success” if the TCS on Day 45 (study exit) was a 2 or less, otherwise the case was classified a ‘failure’; all animals that withdrew from the study early for a treatment related reason were also classified as failures regardless of their TCS.

In addition, TCS and RTS were compared between visits Day 0 and Day 45; dogs that did not return for follow-up visits, those with major protocol deviations or those that did not have positive cultures for qualifying pathogens on Day 0 were not included in such analyses. In this instance, missing data from treated dogs that had to exit the study prematurely were replaced using a “*last observation carried forward* (LOCF)” rule.

The RTS on Day 45 was analyzed using SAS PROC GLIMMIX with the fixed effects of ‘Treatment’ and one of the following: ‘History of Otitis’, ‘Breed’, ‘Weight’, ‘Type of Pathogen’ and ‘Recurrent Status of Otitis’, separately, ‘Site’ and ‘Treatment by site’ were included as random effects in each model. The logit link function and VC covariance structure were utilized for each analysis.

Changes in body weight compared to pretreatment values were analyzed using an analysis of variance (ANOVA; SAS PROC MIXED) including the same effects as above. Treatment groups were compared using LSMEANS.

Hematology, serum chemistry, and urinalysis variables were statistically evaluated using an analysis of covariance (ANCOVA; SAS PROC MIXED) with the pre-treatment value used as a covariate. The model included the same effects as for efficacy above. Prior to performing the ANCOVA, Levene’s test for homogeneity of variance (SAS PROC GLM) was performed. If the results from the Levene’s test were not significant, untransformed data were used in the ANCOVA. In other cases, the data were transformed beforehand (e.g. logarithmic, square root, or reciprocal transformation).

For all statistical analyses, the level of significance was set at *p* = 0.05 for two-sided analyses.

## Results

### Participant flow

**Fig. 1:**
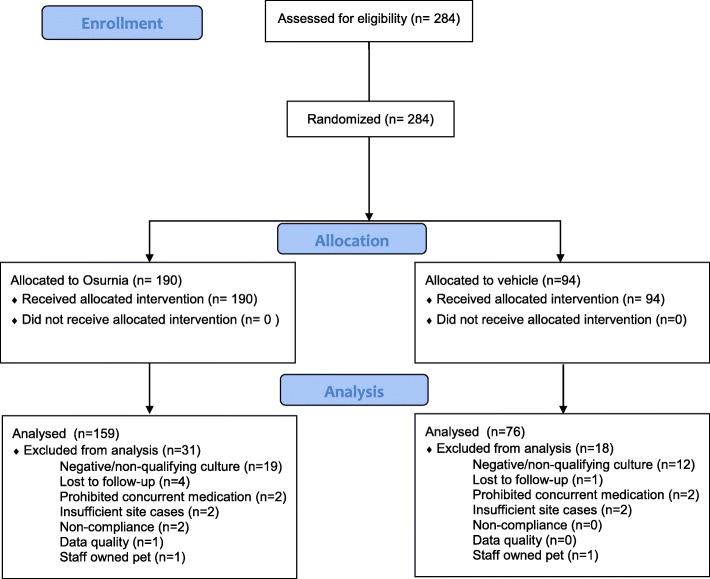
Case flow diagram

In total, 284 dogs from 49 different breeds were enrolled between April and July 2012. They were randomized to receive either Osurnia (190) or its vehicle control (94). While all randomized cases were included in safety analyses, 49 cases were excluded leaving 235 (159 Osurnia, 76 vehicle) cases for treatment efficacy evaluation. The reasons for exclusion of these dogs included: lost to follow-up (*n* = 5), use of a forbidden concurrent medication (*n* = 4), a negative or non-qualifying culture at enrollment (*n* = 31), an insufficient number of cases enrolled at the site (*n* = 4), a significant delay in presentation for the final visit (*n* = 2), data quality (*n* = 1) and staff-owned pets (*n* = 2). One dog was not evaluated on Day 14; while the missing time point was excluded, the case remained for efficacy evaluation on Day 45 (Fig. 1).

Altogether, 64/284 of the total dogs enrolled (23%) exited the trial early primarily due to lack of efficacy (*n* = 51) followed by adverse events (*n* = 8) and loss to follow-up (*n* = 5). These early exits represented 31/94 (33%) of the dogs with ears treated with placebo and 33/190 (17%) of those with an ear treated with Osurnia. Early exit related to adverse events were similar between the two groups.

### Baseline data

On Day 0, before treatment, the two treatment groups had similar descriptive statistics in regards to their age, body weight, gender, number of ears affected, history of otitis, exudate type, and frequency of otitis or presence of subjective hearing loss (Table 2).

**Table 2:** Comparison of treatment groups at baseline

		Osurnia	Placebo	*P*-Value
Number		190	94	
Age	(mean (range); yrs	6.1 (0.3–16.4)	6.1 (0.3–15.8)	0.9097
Body weight	< 40 lbs.; N (%)	78 (41%)	41 (44%)	0.8617
	40–79 lbs.; n (%)	64 (34%)	32 (34%)	
	≥80 lbs.; n (%)	48 (25%)	21 (22%)	
Gender	F:M ratio	90:100 = 0.9	52:42 = 1.2	0.2564
Ears affected	One ear; n (%)	17 (9%)	7 (7%)	0.8217
	Two ears; n (%)	173 (91%)	87 (93%)	
History of otitis	Acute; n (%)	47 (25%)	29 (31%)	0.5436
	Subchronic; n (%)	73 (38%)	32 (34%)	
	Chronic; n (%)	70 (37%)	33 (35%)	
Exudate Type	Erythematous/ceruminous; n (%)	158 (83%)	78 (83%)	1.000
	Suppurative; n (%)	32 (17%)	16 (17%)	
Frequency of Otitis	Recurrent otitis; n (%)	22 (12%)	9 (10%)	0.6894
Hearing Loss	Can hear at Day 0; n (%)	181 (97%)	90 (96%)	0.4846

In both groups, the range of ages and breeds (not shown) was equally variable and males and females were similarly represented. Dogs with bilateral erythematous and ceruminous OE predominated. The OE was considered recurrent in only 11% of cases. Nearly all dogs (> 96%) were subjectively assessed as hearing properly at baseline.

The prevalence of microorganisms cultured from ear swabs obtained on Day 0 were *Malassezia pachydermatis* (70% of samples), *Staphylococcus pseudintermedius* (46%), *Pseudomonas aeruginosa* (11%), beta-hemolytic *Streptococcus* spp. (10%), *Escherichia coli* (6%) and *Proteus mirabilis* (4%)*; Candida* species were not cultured.

### Efficacy outcome measures

**Fig. 2:**
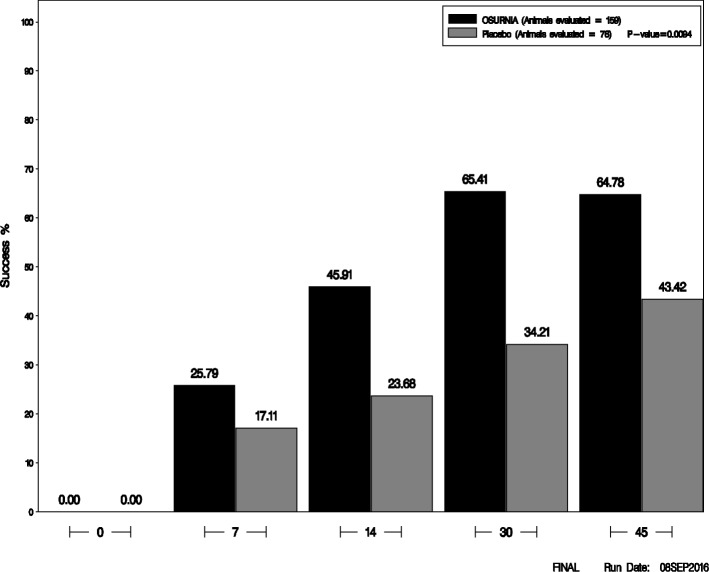
Treatment success rate by treatment group for each study day

A total of 235 dogs (159 treated with Osurnia, 76 with placebo) were included in the analysis of efficacy. In dogs from both treatment groups, the mean TCS—as well as that of each of its components (pain, erythema, exudate, swelling, odor and ulceration)—decreased progressively over time until Day 30, and they generally either plateaued or increased slightly between Day 30 and Day 45 (Fig. 2).

The primary outcome measure of efficacy, the RTS (i.e. a TCS of 0, 1 or 2 on Day 45), was 103/159 (64.78%) for ears treated with Osurnia and 33/76 (43.42%) for ears treated with placebo; this RTS at study end was significantly different between groups (*p* = 0.0094).

The RTS gradually improved after the first dose of Osurnia administered until Day 30, when it plateaued until Day 45 (Fig. 1). In ears treated with the placebo vehicle, the RTS also improved progressively and regularly until the end of the study, but in a proportion always lower to that of ears treated with Osurnia. As would be expected, the RTS decreased with the type of history of OE: ears with acute, subchronic or chronic otitis treated with Osurnia had a respective RTS of 85, 66 and 52% on Day 45. However this was numerically better than those treated with the placebo which had a respective RTS of 44, 52 and 36% on Day 45, respectively.

There was no significant interaction between treatment efficacy and the following variables: duration of history of otitis externa (i.e. acute, subchronic or chronic; *p* = 0.208), recurrence of OE (*p* = 0.731) or body weight (*p* = 0.904). Because of lack of convergence, the statistical model used did not permit the comparison of treatment effect when breed or microbial pathogen types were included as possible cofactors.

### Minimum inhibitory concentrations

The minimum inhibitory concentration (MIC)_50/90_ for florfenicol and terbinafine were calculated; a summary of these MICs for pathogens found in at least 10 isolates is detailed in Table 3.

**Table 3:** MIC_50_ and MIC_90_^a^

Bacteria genus/species	# isolates tested	MIC_50_ (μg/mL FFC)	MIC_90_ (μg/mL FFC)
*Escherichia coli*	25	16	16
*Proteus mirabilis*	17	8	8
*Pseudomonas aeruginosa*	52	> 64	> 64
*Staphylococcus pseudintermedius*	170	4	4
*Streptococcus spp*	52	2	2
Yeast genus/species	# isolates tested	MIC_50_ (μg/mL TRB)	MIC_90_ (μg/mL TRB)
*Malassezia pachydermatis*	239^b^	0.015	0.06

^a^These data represent all 392 isolates (284 from Day 0 and 108 from treatment failure cases)

^b^Twenty-one *M. pachydermatis* samples tested did not grow in the MIC plates

Overall, and including all isolates from all visits, the florfenicol MIC was lowest for *Streptococcus* spp. (MIC_90_ = 2 μg/mL) and *Staphylococcus pseudintermedius* (4 μg/mL), intermediate for *Proteus mirabilis* (8 μg/mL) and *Escherichia coli* (16 μg/mL), and highest for *Pseudomonas aeruginosa* (> 64 μg/mL). The overall terbinafine MIC profile for *Malassezia pachydermatis* isolates ranged from ≤0.004–0.25 μg/mL with an MIC_90_ of 0.06 μg/mL. Importantly, the MIC profiles of baseline isolates were nearly identical to those collected from ears evaluated as treatment failures with only a single doubling dilution change in the MIC_90_ of the yeast.

### Safety evaluation

All 284 enrolled dogs (190 Osurnia treated, 94 placebo control) were considered for clinical safety. The most frequently reported adverse reactions with possible relationship to treatment were elevated clinical chemistry values and vomiting. Reported adverse reactions with possible relationship to treatment are presented in Table 4.

**Table 4:** Adverse reactions

Adverse reaction^a^	Osurnia		Placebo	
	N	% of total (*n* = 190)	N	% of total (*n* = 158)
Elevated Alkaline Phosphatase	15	7.9	3	3.2
Vomiting	7	3.7	1	1.1
Elevated AST, ALT, ALP^a^	2	1.1	0	0.0
Weight Loss (10% BW)	1	0.53	0	0.0
Hearing Decrease/Loss	1	0.53	1	1.1

^a^Aspartate aminotransferase (AST), alanine aminotransferase (ALT), alkaline phosphatase (ALP). Two dogs with pre-existing elevations in ALP were reported to have an increase in liver enzymes (ALP, ALT and/or AST) at study exit. Subsequent clinical chemistries returned to normal

However, skin and appendage disorders such as pruritus and dermatitis were the overall most frequently reported AEs noted during the study. These likely represented pre-existing allergic skin diseases.

There was no detectable association between breed, age, gender or number of doses received and the development of AEs. Importantly, there was no evidence of increased irritation (i.e. pain, erythema, swelling or ulceration of the ear canal) associated with dosing of Osurnia.

One dog in each treatment group was reported by the owner as having hearing impairment at the Day 14 visit. In neither case could the hearing loss be confirmed in clinic and all dogs treated with the active product on Day 0 could hear on Day 45, or at the visit scheduled for early study exit.

Finally, there were no significant changes in body weight, hematology, serum chemistry and urinalysis variables between dogs treated with Osurnia or placebo (data not shown); in all cases, means remained within the normal range.

## Discussion

This RCT demonstrates the efficacy and safety of the application of Osurnia, at two doses one week apart, to treat canine OE due to the common microbial pathogens classically seen in this syndrome. The adaptable gel formulation requires less frequent product administration than typically approved OE therapies and, other than cleaning prior to the first administration, does not necessitate the need for additional ear cleaning between treatments which is typically recommended for OE treatment [3, 8].

### Generalizability of the results

Overall, the dogs enrolled are representative of general practice patients with microbial OE that are the most likely to be treated with Osurnia. The subjects included exhibited typical characteristics: there was a wide range of breeds, ages and weights and both genders were equally represented. Cases enrolled had an acute and subchronic history more often than chronic OE, and the exudate was more often erythematous and ceruminous rather than suppurative; nearly all dogs appeared able to hear at the beginning of the study. At baseline, microbial cultures most often grew *Malassezia* yeast and *Staphylococcus pseudintermedius*, which are the two most common pathogens isolated in canine OE [3].

The number of patients included was not a source of imprecision, as the results obtained post-trial were compatible with the model estimated at the time of sample size calculation. The efforts made to mask the nature of treatment and to blind personnel and owners proved to be sufficient to avoid unblinding and subsequent bias in treatment efficacy assessments.

In this trial, the clinical assessment scale (TCS) and the primary outcome measure (RTS) were both considered appropriate for assessment of OE as they are based on clinical signs that are relevant to both dog owners and veterinarians. The TCS was established using six classic OE signs/symptoms, each with a maximum of two points, giving a maximum total of 12 points. The RTS, defined as a TCS of 0, 1 and 2, represents an ear canal with no or little residual signs of OE at study end, i.e. an ear likely to be perceived as “*cured*”. Requiring a minimum TCS of at least 6 points for enrollment in the study ensured cases with an OE of at least moderate severity were included in the study. If we take into consideration that the mean TCS of patients at enrollment was 8/12, on average, successful cases had an improvement of at least 6 points (or 50%) over a 12-point scale. Therefore, the primary outcome measure used in this study (RTS) should be considered clinically relevant.

### Interpretation of efficacy

In this trial, the effectiveness of Osurnia demonstrated superiority to that of placebo, as the RTS at study end was significantly higher in dogs treated with the active product than those treated with the placebo. In addition, all the individual components of the TCS for the Osurnia treated animals showed improvement over time. It is not surprising that the weakest treatment effect was observed for the parameter “*exudate*”. Exudates need to be differentiated from the ubiquitous cerumen seen in canine ears and in this study, the use of traditionally recommended ear cleaning in cases of microbial OE was not performed thereby potentially impacting this score [8].

For the primary outcome measure, the number-needed-to-treat (NNT, the inverse of the RTS difference between groups) could be calculated as 5 (i.e. five dogs would need to be treated with Osurnia to obtain one more success compared to placebo). This seemingly elevated NNT is not due to the low proportion of RTS in the actively treated group, but due to the unusually high effect of the placebo vehicle. Indeed, the mean TCS and each of its components fell rapidly after the second application of Osurnia and placebo. The return to apparent normalcy in the subjectively evaluated “*pain*” symptom suggests that not only Osurnia, but also the vehicle itself, might have some antalgic effect; this effect might follow, or be concurrent to, the mild anti-inflammatory effect seen on erythema and swelling with both interventions. It can be speculated that ear cleaning to remove debris and exudate prior to treatment administration may be partially responsible for the apparent treatment response in placebo treated dogs, leading to some dogs apparently self healing [8]. However, other possible causes, such as antimicrobial activity of the placebo itself could be considered [10, 11]. This possible activity of the vehicle alone was unexpected, and it is deserving of further evaluation.

In this study, the determination of the MICs for florfenicol confirmed the variability of bacterial susceptibility to this antibiotic, being highest for the Gram negative rods, especially *Pseudomonas aeruginosa*. As a consequence, the efficacy of florfenicol-containing topical ear formulations might be expected to be lower in cases of *Pseudomonas* OE, especially whenever this antibiotic concentration does not exceed the MIC for the organism; however it may be noted that antibiotics, when applied topically rather than given systemically, will more often than not exceed the MICs [12]. Importantly, the bacterial MICs from OE cases failing to respond were not different from those seen at baseline indicating that the treatment failures in dogs administered Osurnia were not due to selection of resistant microbial isolates.

### Interpretation of safety

In this RCT, the two doses of Osurnia or its vehicle were well tolerated. Importantly, the overall most commonly reported non-serious AE were pruritus and dermatitis, which likely represented primary factors relating to development of OE rather than resulting from treatment. There were no detectable changes in body weights and clinical pathology parameters (except ALP) during this study and as such would preclude the need for any specific monitoring of these parameters when administering Osurnia. Although a greater number of cases treated with Osurnia had elevated ALP these were not considered to be of clinical significance. These ALP increases were not reported as AEs during the study, remained within normal clinical range and represented less than a 2–3 fold increase. Similarly for vomiting, although a greater number of dogs in the Osurnia treated group vomited, in no case was it considered to be clinical relevant or related to treatment according to the investigators. Importantly, Osurnia used under the conditions of this trial did not lead to any detectable change in response to a clap test, although it is recognised that only Brainstem Auditory Evoked Response (BAER) can truly assess loss of hearing [13]. The post-marketing experience in the EU suggests that deafness or impaired hearing may be a very rare (less than 1 animal in 10,000 treated) event, mainly in elderly animals [14].

### Limitations

There were several possible limitations resulting from the design and/or performance of this RCT.

Firstly, there was an initial loss of 49/284 dogs (17%) due to exclusion for a variety of reasons, the main one being the lack of growth of qualifying pathogens at the study onset. Furthermore, one fifth of dogs that began treatment had to exit the study early, mostly due to lack of efficacy. This attrition rate was not likely a source of bias as the early exits for lack of efficacy were higher in placebo (29%) compared to Osurnia (14%) treated animals which would be expected.

Evaluation systems, similar to TCS as an assessment scale and RTS as a primary outcome measure, have been used consistently for approval of other OE treatments with the FDA [15–17], as there are no universally accepted and validated clinical scoring systems for OE. As such use of these could be perceived as not validated which already has been mentioned as a possible source of detection bias in a systematic review of interventions to treat *Pseudomonas* OE [18]. In spite of this limitation, the parameters subjectively evaluated in this study are clinically meaningful and comparable to those mentioned in a recent paper reviewing OE severity scales [19]. Furthermore, the primary outcome measure was chosen to be a near return to normalcy, which should help alleviate any possible detection bias.

The lack of performance of an otic cytology before and after treatment could lead to the perception that not all cases assessed as successfully treated were indeed so. That the main outcome measures focussed on clinical signs rather than cytology was deemed preferable as, even though cytology is perceived to be the “*gold standard*” for OE assessment, it is far from capturing the complex reality of the skin and ear microbiome of normal and allergic dogs [20, 21]. Although cytology and clinical assessment are generally correlated in dogs with OE [22], it has not been possible to establish thresholds with sufficient sensitivity and specificity to allow the use of cytological endpoints in clinical studies [23]. Additionally, in this study, microbial culture was done before treatment, and culture and cytology results have been shown to generally correlate in dogs with either *Malassezia* [23] or microbial OE [9].

Another limitation is that, to be entered in this study, dogs had to have tympanic membranes visible by otoscopy before treatment. This selection criterion, which was proposed as a precaution to limit any potential drug-induced ototoxity, clearly eliminated cases with any hyperplastic ear canals, as seen in dogs with very chronic OE. Even though we did observe that the RTS of dogs with a history of chronic OE was one third lower than that of dogs with acute OE, the factor “*duration of history of OE*” was found not to affect treatment outcome.

The statistical model used did not allow for the determination of any possible influence of the type of microbes cultured and the RTS; this likely reflecting the complexity of OE and its associated microbiome. The study was not specifically designed or powered to answer this question, limiting the determination of which type of microbial OE best responds to the application of Osurnia.

## Conclusion

Because of its treatment success rate and minimal clinical safety findings, Osurnia formulated as an adaptable gel, applied twice, one week apart, is a valuable addition in the armamentarium against this common syndrome affecting dogs including first line cases. The ease of use brought by the gel formulation which remains at the site of application and its infrequent dosing are two important additions that should help clients better comply with the veterinarian’s prescription.

## Abbreviations

AE: Adverse Event
ALP: Alkaline Phosphatase
ALT: Alanine Aminotransferase
ANOVA: Analysis of Variance
ASP: Aspartate Aminotransferase
FFC: Florfenicol
LOCF: Last Observation Carried Forward
MIC: Minimum Inhibitory Concentration
NNT: Number Needed to Treat
OE: Otitis Externa
RCT: Randomized Controlled Trial
RTS: Rate of Treatment Success
TCS: Total Clinical Score
TRB: Terbinafine
VC: Variance Components
